# The role of an objective function in the mathematical modelling of wide-angle X-ray diffraction curves of semi-crystalline polymers

**DOI:** 10.1107/S2053273321007762

**Published:** 2021-09-24

**Authors:** Małgorzata Rabiej, Stanisław Rabiej

**Affiliations:** a Faculty of Materials, Civil and Environmental Engineering, University of Bielsko-Biala, Willowa 2, Bielsko-Biała, 43-309, Poland

**Keywords:** polymers, diffraction curves, absolute error, relative error, objective function, mathematical modelling

## Abstract

To decompose a wide-angle X-ray diffraction (WAXD) curve of a semi-crystalline polymer into crystalline peaks and amorphous halos, a theoretical best-fitted curve, *i.e.* a mathematical model, is constructed. In fitting the theoretical curve to the experimental one, various functions can be used to quantify and minimize the deviations between the curves. The analyses and calculations performed in this work have proved that the quality of the model, its parameters and consequently the information on the structure of the investigated polymer are considerably dependent on the shape of an objective function. It is shown that the best models are obtained employing the least-squares method in which the sum of squared absolute errors is minimized. On the other hand, the methods in which the objective functions are based on the relative errors do not give a good fit and should not be used. The comparison and evaluation were performed using WAXD curves of seven polymers: isotactic polypropyl­ene, polyvinylidene fluoride, cellulose I, cellulose II, polyethyl­ene, polyethyl­ene terephthalate and polyamide 6. The methods were compared and evaluated using statistical tests and measures of the quality of fitting.

## Abstract 2

The choice of an objective function in constructing a mathematical model of an experimental wide-angle X-ray diffraction curve of semi-crystalline polymers is discussed.

## 1. Introduction   

The wide-angle X-ray diffraction (WAXD) curve of a semi-crystalline polymer gives important and unique information on its internal structure. By analysing such a curve we can determine the unit-cell parameters, the degree of crystallinity, the size of crystallites, the degree of orientation, the weight fractions of polymorphic phases. Moreover we can investigate the influence of different factors on all these data.

To perform these investigations and analyses, the intensity contributions arising from crystalline and amorphous regions of the polymer have to be isolated and separated from each other. It means that we have to decompose the WAXD curve into individual constituents. To this aim a theoretical curve is constructed which is a best-fitted mathematical model of the experimental one (Russell *et al.*, 1993 ▸, 1997 ▸; Hu & Hsieh, 1996 ▸; Chen & Yokochi, 2000 ▸; Sajkiewicz *et al.*, 2005 ▸). The theoretical curve is described by a function which is a sum of component functions related to individual crystalline peaks, amorphous halos and background scattering. Each component function related to a crystalline peak or amorphous halo has four parameters at least: angular position, height, width at half-height and so-called shape coefficient (Hindeleh & Johnson, 1974 ▸, 1978 ▸). The number of peaks, their shape and size depend on the polymer type, its crystalline structure and crystallization conditions.

Because of the large number of unknowns, the multidimensional space of solutions and the diversified shape of the functions which describe the crystalline and amorphous components, the fitting procedure and determination of all parameters is one of the most difficult non-linear optimization tasks. As has already been shown (Rabiej, 2003 ▸, 2014 ▸; Rabiej & Rabiej, 2005 ▸) the solution of this task can be made much more unambiguous and reliable when we employ a bi-criterial optimization procedure. It involves not only the best fitting of the theoretical curve to the experimental curve, *i.e.* minimization of the deviations between the curves, but also simultaneous maximization of the area of amorphous component. The latter criterion results from observations of the early stages of crystallization of polymers in dynamic synchrotron investigations (Bark *et al.*, 1992 ▸; Wang *et al.*, 2000 ▸). This criterion ensures a smooth transition from the WAXD curves of a molten polymer to the curves in which first traces of crystalline peaks start to be visible. The two criteria of the optimization procedure have different roles. The best fitting of the curves is the superior, dominating and final condition. The second criterion has a steering role and gives a direction in which the space of solutions has to be searched. Therefore the shares of the two criteria must be represented by suitable weights (Rabiej, 2017*b* ▸). Experimental practice has shown that the classical optimization methods are not effective in performing bi-criterial optimization. This is why new methods employing artificial intelligence have been elaborated; they are much more effective, faster to converge and lead to globally best solution procedures (Rabiej, 2013 ▸, 2014 ▸). Recently, a procedure based on the particle swarm optimization (PSO) method (Kennedy & Eberhart, 1995 ▸) has been proposed (Rabiej, 2017*a* ▸).

In this work it is shown that the quality of the mathematical model of an experimental WAXD curve is clearly dependent on the shape of objective function which is minimized in the fitting procedure. To this end we have compared the models of the WAXD curves obtained using two methods in which the absolute errors are minimized (the least-squares method and the least absolute deviations method), and four methods where the relative errors are minimized (least-squared relative deviations method, least absolute relative deviations method, least relative squares method and weighted least-squares method). The comparison and evaluation were performed using WAXD curves of popular polymers. The methods were compared and evaluated using statistical tests and measures of the quality of fitting.

## 2. Experimental   

To compare and evaluate the methods of fitting mentioned above, the WAXD curves of seven polymers were recorded. The WAXD curves of the chosen polymers have clearly different shapes: isotactic polypropyl­ene (iPP), polyethyl­ene (PE) and polyvinyl­idene fluoride (PVDF) with several high and narrow crystalline peaks, cellulose II (CeII) and polyamide 6 (PA6) with broad and not very high peaks, and finally cellulose I (CeI) and polyethyl­ene terephthalate (PET) with both sharp and broad crystalline peaks.

The investigations were performed by means of a URD-6 Seifert (Germany) diffractometer using a symmetrical reflection mode and a copper target X-ray tube (λ = 1.54 Å) operated at 40 kV and 30 mA. The Cu *K*α radiation was monochromatized with a graphite monochromator. For each polymer the WAXD curves were recorded in the same 2θ range 5–60° with a step size of 0.1° and the same registration time: 20 s step^−1^. The isotropic samples were prepared using fibres made of the investigated polymers which were powdered by means of a microtome and pressed into a sample holder. The obtained samples had the shape of circular pills with a diameter of 2 cm and thickness of 1 mm.

Before the main calculations a linear background scattering composed of the incoherent Compton scattering, air scattering, thermal diffuse scattering and radiation from external sources was subtracted from the curves and all curves were normalized in such a way that the total area under each curve was equal to unity.

The curves were decomposed into crystalline peaks and amorphous components using a bi-criterial PSO method (Kennedy & Eberhart, 1995 ▸). The PSO algorithm is an artificial intelligence method. Its action imitates the behaviour of a bird flock or a fish school. Instead of using one starting set of parameters, this algorithm creates several tens of such sets by a draw. In this way some population of solutions is created. The population of candidate solutions is considered as a swarm and individual solutions are called particles. In the successive steps, particles move to new places, simulating adaptation of a swarm to the environment. As the population is created by a draw, the probability that the globally best solution will be found is much higher than in the classical optimization methods. Because the size and direction of the search step are dependent on the best solution found at a given stage of action, the algorithm is much less dependent on the starting parameters. In the system used in this work a few modifications have been introduced to the original PSO method (Rabiej, 2017*a* ▸). As a result, it is better adapted for decomposing the WAXD curves.

Starting values of the angular positions of crystalline peaks were determined based on the respective unit-cell parameters of the investigated polymers. The crystalline peaks and amorphous maxima were approximated by a linear combination of Gauss and Cauchy profiles.

Each curve of a given polymer was decomposed employing five tested methods (M1, M2, M3, M4 and M6) described in Section 4, implemented in the program *WAXSFIT*. To employ and evaluate the weighted least-squares method (M5), the diffraction curves of four polymers (iPP, PVDF, CeI and CeII) were recorded five times using the same experimental conditions.

Each decomposition procedure using a given method resulted in one mathematical model of the curve. The quality of models (*i.e.* the quality of fitting) was evaluated using the measures and tests described in Section 5, implemented in the *WAXSFIT* program as well. For a given curve and a given method, the decomposition procedure was repeated ten times using different starting parameters. Based on ten results of decomposition, the average value and standard deviation for each parameter of a theoretical curve were calculated.

Furthermore, the final values of the information criteria and statistical tests used for evaluation of the employed methods of fitting were calculated by averaging the results obtained in those ten runs.

All obtained data were elaborated using the computer program *WAXSFIT* (Rabiej, 2017*a* ▸).

## 3. Statistical evaluation of errors in a WAXD curve recording   

When a diffraction curve is recorded, the number of impulses representing a local intensity *y_i_* of X-rays scattered at a given diffraction angle *t_i_* (2θ) is counted. The angle is changed with a constant step in some arbitrarily assumed range. As a result we obtain a set of values (*y*_1_, …, *y_n_*) related to the subsequent diffraction angles (*t*_1_, …, *t_n_*). Each individual result *y_i_* can be considered as one possible value of a random variable *Y_i_*. This result is characterized by some measurement uncertainty (random error) and should be treated as an approximation of the real value, represented by the mathematical expectation E(*Y_i_*) of the random variable *Y_i_*. Later on, the mathematical expectation, *i.e.* the theoretical value of a model, will be denoted as 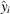 in this paper.

The absolute error and relative error are defined by equations (1) and (2), respectively:

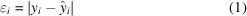




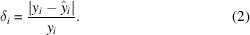

Generally, the uncertainty of measurements results from the equipment limitations and measurement conditions. The intensity recorded at a given angle is the sum of a component related to the structure of the investigated sample and the background scattering. The latter component comprises incoherent Compton scattering, air scattering *etc*. and must be subtracted from the experimental curve before the mathematical model of the curve is constructed. Usually the diffraction curve for a given sample is recorded once. So, the mathematical expectations 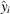 are not known and consequently neither absolute nor relative errors can be calculated. A single measurement says nothing about the random value *Y_i_* distributions, their standard deviations σ_*i*_ and mathematical expectations 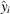 .

When the number of counts is small, the probability that the result of a single measurement amounts to *y_i_* can be described by the Poisson distribution (Goldanski *et al.*, 1963 ▸)

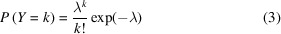

where *k* = *y_i_* and λ is the mathematical expectation λ = E(*Y_i_*) = 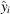 .

The deviation of a random value from its mathematical expectation is described by its variance and standard deviation, *i.e.* the square root of the variance. In the Poisson distribution the variance σ^2^ is equal to the mathematical expectation λ, thus

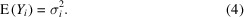

This means that the standard deviation 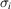 of each result of a single measurement *y_i_* is equal to the square root of the mathematical expectation 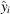 :

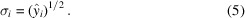

As the mathematical expectation is not known 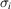 can be substituted by its estimator, standard error SE_*i*_:

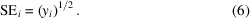

Consequently, the bigger the number of counts *y_i_*, the smaller is the relative uncertainty of the measurement:

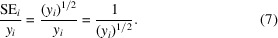

According to the central limit theorem (Ash & Doleans-Dade, 2000 ▸), when the number of counts increases (*k* → ∞) the Poisson distribution transforms smoothly into the Gauss distribution *N*(*m*, σ), with the mathematical expectation *m* and standard deviation σ. So, if the Gauss distribution approximates the Poisson distribution, equations (6) and (7) are still valid.

The mathematical expectations ( 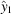 , 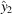 , …, 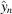 ) of the number of impulses representing local intensities at subsequent diffraction angles (*t*_1_, …, *t_n_*) can be determined in two ways. The first method consists of sequential recording of diffraction curves for a given sample. The second method consists of the creation of a mathematical model of the curve.

### 3.1. First method   

In this case, to obtain a credible estimation of the mathematical expectations 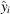 , a diffraction curve of a sample has to be recorded several times (*N*) at exactly the same experimental conditions. Next, the average number of counts 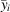 [equation (8)] and root-mean-square error *s_i_* [equation (9)] for each diffraction angle *t_i_* (*i* = 1, …, *n*) have to be calculated:

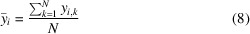




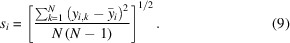

According to the law of large numbers (classical Kolmogorov’s SLLN) (Dekking, 2010 ▸), the obtained values are estimators of the mathematical expectations 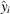 and standard deviations 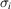 , respectively.

### 3.2. Second method   

The mathematical model of a diffraction curve should ensure that the differences between the experimental *y_i_* and theoretical 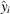 values of intensity at the subsequent diffraction angles (*t*_1_, …, *t_n_*) (*i.e.* the residuals) are as small as possible.

At the first stage of modelling, the functions describing crystalline peaks and amorphous halos are chosen, and the number of optimized parameters is established. Next, the optimization method is chosen. In all optimization methods the objective function is defined which quantifies the differences between the experimental and theoretical values of intensity. Moreover, the set of starting values of optimized parameters and the criterion of accomplishment of the procedure must be provided.

In the successive steps (iterations) of an optimization procedure, the values of parameters are changed in such a way that the value of the objective function becomes lower and lower. The optimization procedure is finished when such a set of parameters is found for which the objective function reaches its minimum.

Let us analyse the influence of absolute and relative errors on the value of objective functions in an optimization procedure.

**Figure 1:**
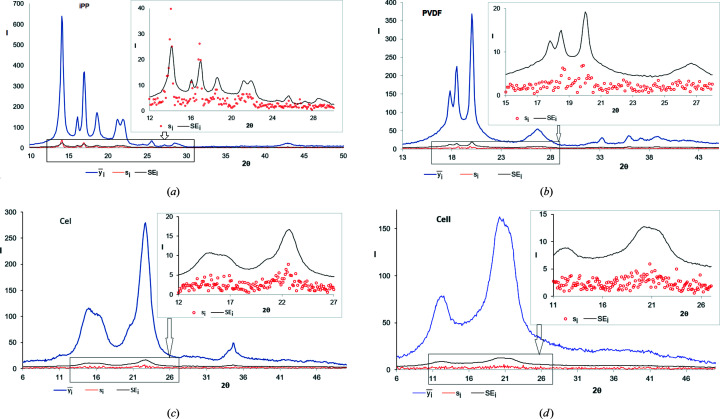
WAXD curves for (*a*) iPP, (*b*) PVDF, (*c*) CeI and (*d*) CeII; navy blue line, 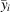 average intensity calculated based on five diffraction curves independently recorded for each polymer; red line and circles, root-mean-square error *s_i_*; black line, standard error SE*_i_*; inset, a magnified fragment of the plot indicated by an arrow.

Figs. 1 ▸(*a*)–1 ▸(*d*) show the average intensity values 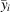 , root-mean-square errors *s_i_* [equation (5)] and standard errors SE_*i*_ [equation (6)], for iPP, PVDF, CeI and CeII samples, calculated based on five diffraction curves recorded for each polymer. As one can see, the values of the root-mean-square error *s_i_* are more or less proportional to the average intensities 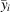 , and they are clearly lower than those of the standard error SE_*i*_. Only at a few points, where the intensity is very high, is the *s_i_* bigger than SE_*i*_.

**Figure 2:**
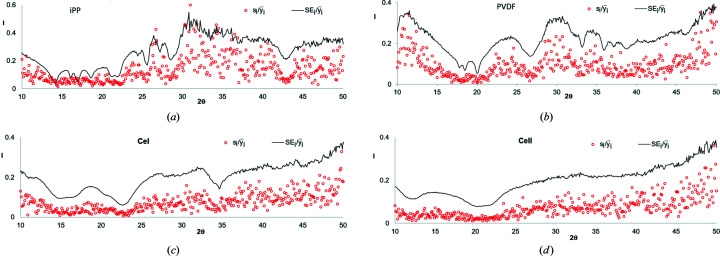
Relative errors 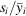 (red circles) and 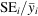 (black line), calculated based on data presented in Fig. 1 ▸ for (*a*) iPP, (*b*) PVDF, (*c*) CeI and (*d*) CeII.

Figs. 2 ▸(*a*)–2 ▸(*d*) show the relative errors, *i.e.* the ratios of the root-mean-square error *s_i_* and standard error SE*_i_* to the average intensity 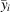 . It is seen that the relative errors in the range of the diffraction angle where high peaks are localized (*t_i_* ≃ 15–25°) are much smaller than those in the range where no peaks are present (*t_i_* ≃ 10–15°and 30–50°). Of course, this is compatible with equation (7), according to which the higher the intensity, the lower the relative error.

The presented figures indicate that when the objective function is created based on the absolute errors, its value is mostly dependent on the points from such ranges of WAXD curves where the crystalline peaks are located. In contrast, when the relative errors are taken into account, the biggest influence on the objective function is from ranges where no peaks are present.

Thus, in order to obtain the highest quality of fitting in the ranges comprising the crystalline peaks we should choose the first solution. On the other hand, if we assume that the quality of fitting is most precisely described by the relative errors, the second possibility should be chosen. In this paper the models obtained with these two types of objective functions are compared and evaluated.

## 4. Methods of fitting a theoretical curve to the experimental WAXD curve   

In the bi-criterial optimization procedure the first requirement for optimal fitting of a theoretical curve to the experimental WAXD curve is the superior one. It can be reached by means of different methods of fitting using different objective functions *F* which are minimized to obtain the best quality of fitting. The methods analysed and compared in this work are listed below.

*M1. Least-squares method*. The method of least squares is one of the fundamental tools in scientific investigation (Nielsen, 2000 ▸, 2001 ▸). It consists of minimization of the sum F1 of squared deviations between experimental intensities *y_i_* and theoretical ones 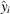 resulting from a mathematical model of the WAXD curve,

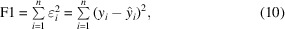

where *n* is the number of experimental points (diffraction angles) and 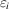 is a random error.

This method assumes that the experimental measurements are free of systematic errors and are performed with a random, normally distributed error with the same variance at each diffraction angle. The least-squares method is very sensitive to the local outliers, *i.e.* single atypical intensity values, considerably different from the remaining ones, which are caused by various random factors.

*M2. Least absolute deviations method*. This method is less sensitive to the negative influence of outliers on the obtained parameters of the model. It consists of minimization of the sum F2 of absolute deviations between experimental and theoretical intensities,

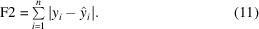




*M3. Least relative squares method*. This method takes into account that the biggest standard deviations of intensity values in WAXD curves are frequently observed in such ranges of diffraction angle where strong crystalline peaks are located. This is why the squared deviations between experimental and theoretical intensities at a given diffraction angle are divided by the local variance. In this way a lower weight is attributed to the less accurate measurements and a higher weight to the more precise ones (Strutz, 2016 ▸).

As the variance 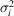 is not known, it is substituted by its estimator – standard error 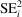 . Thus, the minimized objective function F3 has the shape of equation (12):

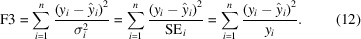




*M4. Least absolute relative deviations*. The method is similar to the previous one but this time the absolute deviations between experimental and theoretical intensities at a given diffraction angle are divided by the local experimental intensity. The minimized objective function F4 is given by

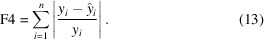




*M5. Weighted least-squares method*. Similar to the method M3, this one also takes into account that the variance of intensity is not the same at all diffraction angles. For this reason, the squared differences between experimental and theoretical intensities at a given diffraction angle are divided by the local variance 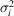 .

To employ this method the diffraction curve of each polymer was recorded five times at the same experimental conditions. Based on these data, the average intensity 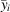 [equation (8)] and root-mean-square error *s_i_* [equation (9)] for each diffraction angle were calculated. Next 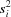 was used as an estimator of the local variance 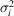 .

The minimized objective function F5 is given by

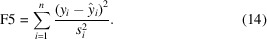




*M6. Least-squared relative deviations method*. In this method the minimized objective function F6 is calculated as the sum of squared deviations between experimental and theoretical intensities divided by the squared experimental intensity at a given diffraction angle. In other words, it is the sum of squared relative deviations, 

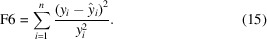




## 5. Measures and statistical tests for evaluation of the fitting methods   

A comparison and evaluation of the methods of fitting described above and obtained mathematical models of an experimental WAXD curve should encompass three aspects.

(i) First of all we have to check if positions of crystalline peaks determined with a given method agree with those resulting from the unit-cell parameters of the investigated polymer.

(ii) Next we should check if residuals, *i.e.* the final values of differences between the experimental and theoretical best-fitted curve, fulfil all conditions required by the methods. To assess credibly the statistical correctness of a model we should check if the residuals fulfil a set of conditions listed below. The residuals should be:

(*a*) Unbiased, *i.e.* the expected value of residuals is zero.

(*b*) Symmetric, *i.e.* the numbers of positive and negative residuals are the same.

(*c*) Random, *i.e.* the experimental points must be randomly dispersed along the theoretical curve.

(*d*) Uncorrelated, *i.e.* a lack of autocorrelation of the residuals – there are no hidden trends in their course.

(*e*) Homoscedastic, *i.e.* the variance of the residuals should be constant.

Moreover, method M1 demands the residuals to be normally distributed.

When the residuals do not fulfil the required conditions, it means that the mathematical model of the curve is wrong and should be rejected.

In this work the first four conditions were checked by the following statistical tests: unbiasedness of residuals test (Schwefel, 1981 ▸; Conover, 1999 ▸), symmetry of residuals test, Wald–Wolfowitz series test (Wald & Wolfowitz, 1940 ▸), Durbin–Watson test (Durbin & Watson, 1951 ▸; Hill & Flack, 1987 ▸). To verify the fifth condition, the whole range of diffraction angles in which the WAXD curves were recorded was divided into subranges and the average values of residuals in the subranges were compared with each other, respectively. The normality of residuals in method M1 was tested by means of the chi-squared test (Greenwood & Nikulin, 1996 ▸).

(iii) Finally, we should assess the quality of fitting, *i.e.* the quality of the obtained model of the curve.

In the literature we found various measures of similarity, so-called information criteria, used for the statistical assessment of the quality of models and for their comparison. Comparing several models, the information criteria help to estimate which one of them is most suitable for a given set of experimental data. In this work two information criteria were employed:

Integral similarity index (Hofmann & Kuleshova, 2005 ▸) *S_s_*:

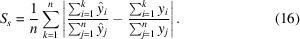




Normalized index (Hofmann & Kuleshova, 2005 ▸) *R*:

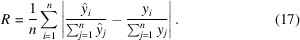

By means of these parameters different models of the same diffraction curve can be compared. More precisely, they allow one to compare an experimental curve with different models of this curve.

The best model is that one for which the information criteria reach the smallest values. Both *R* and *S_s_* are equal to zero when the quality of fitting is the best, *i.e*. the theoretical and experimental curves are identical. It is easy to notice that the integral similarity index *S_s_* is calculated as the average value of the absolute differences between the normalized integral theoretical and experimental intensities. In turn, according to equation (17), the normalized *R* index is the arithmetic average of the normalized absolute differences between the experimental and theoretical curve.

Presenting in one plot the normalized theoretical integral intensity

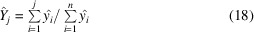

and normalized experimental integral intensity

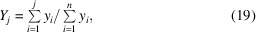

as functions of the diffraction angle, we obtain so-called integral curves which allow for visual evaluation of the quality of fitting. The better the quality of fitting, the closer the theoretical and experimental integral curves are to each other. It is a very sensitive tool for the quality of fitting evaluation (Hofmann & Kuleshova, 2005 ▸).

## 6. Results of investigations and evaluation of the methods   

The WAXD curves of all investigated polymers were decomposed by means of the PSO optimization procedure employing the six tested methods M1–M6. Each decomposition procedure by means of a given method resulted in one model of the curve.

According to Section 5, the evaluation of employed methods was a three-step process. In the first step, the correctness of the angular positions of crystalline peaks determined with the methods was verified. Next, the statistical tests checking if the residuals met the necessary conditions listed in Section 5 were performed and, finally, the quality of the models was assessed.

Before the evaluation procedure the degree of crystallinity (*X*) of the investigated polymers was calculated based on parameters found by means of the compared methods. It was calculated as the ratio of the integral intensity contained in crystalline peaks to the total intensity scattered by a sample. Table 1 ▸ shows the ranges of *X* values obtained with each method in ten runs of calculations performed for the investigated polymers.

The table shows that, depending on the method employed, the results obtained for a given polymer may differ even by several %. As one can see, apart from PET the differences between the crystallinity values determined for a given polymer with a given method amount to several % but the crystallinities obtained with different methods may differ by more then a dozen % from one another, which means that the differences are significant. In the case of PET, the differences are even bigger. This fact clearly confirms that the objective function used in an optimization procedure influences considerably the shape of the theoretical curve and its parameters. This is why the choice of the most credible method is an important task.

### 6.1. Verification of the positions of crystalline peaks   

As concerns cellulose I, cellulose II and PA6 the angular positions of most of the crystalline peaks determined with all six methods agree well with those resulting from the respective unit-cell parameters. Only in the case of small, broad peaks localized at large diffraction angles are the discrepancies slightly higher. The same is true of the curves of iPP, PVDF and PE. Of course, when comparing the angular position of crystalline peaks one should remember that, even in the case of the same polymer but crystallized at different conditions, the positions of peaks may differ slightly. In contrast, in the case of PET, the positions of peaks determined with methods M4 and M6 do not agree with those resulting from the unit-cell parameters.

**Figure 3:**
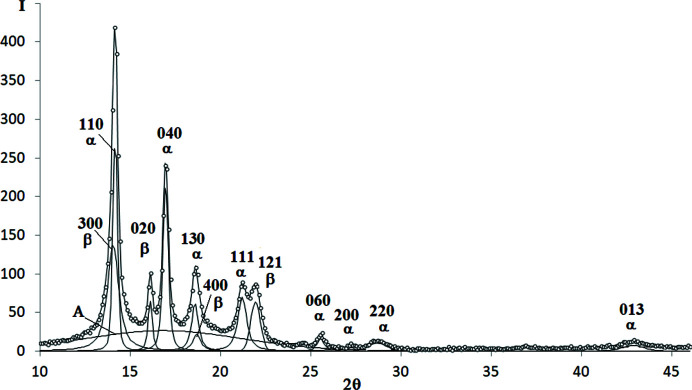
WAXD curve of iPP decomposed into crystalline peaks and amorphous maxima: experimental curve – points, best-fitted theoretical curve and all components – solid line.

The examples of decomposed WAXD curves are shown in Figs. 3 ▸–9 ▸ ▸ ▸ ▸ ▸ ▸ and determined positions of crystalline peaks are collected in Tables 2 ▸ ▸ ▸ ▸ ▸–7 ▸.

*Isotactic polypropyl­ene (iPP)*. The WAXD curve of iPP contains 12 crystalline peaks and two amorphous maxima (Fig. 3 ▸). The total number of unknown parameters determined with the PSO optimization procedures was 56.

**Figure 4:**
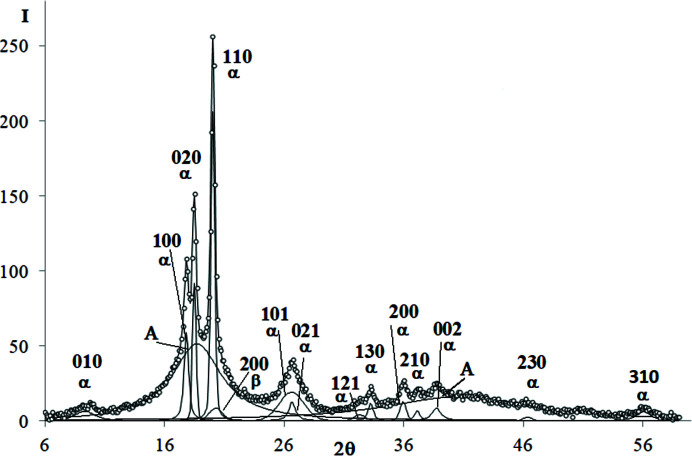
WAXD curve of PVDF decomposed into crystalline peaks and amorphous maxima: experimental curve – points, best-fitted theoretical curve and all components – solid line.

*Polyvinyl­idene fluoride (PVDF)*. The WAXD curve of PVDF contains 14 crystalline peaks and two amorphous maxima (Fig. 4 ▸). The total number of unknown parameters determined with the PSO optimization procedures was 64.

**Figure 5:**
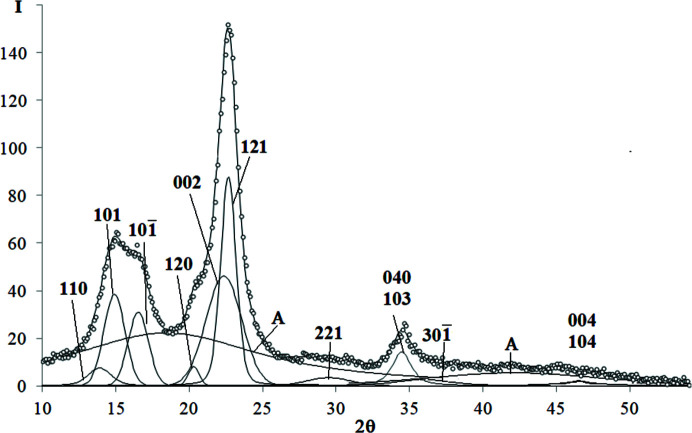
WAXD curve of cellulose I decomposed into crystalline peaks and amorphous maxima: experimental curve – points, best-fitted theoretical curve and all components – solid line.

*Cellulose I (CeI)*. The WAXD curve of cellulose I contains ten crystalline peaks and two amorphous maxima (Fig. 5 ▸). The total number of unknown parameters determined with the PSO optimization procedures was 48.

**Figure 6:**
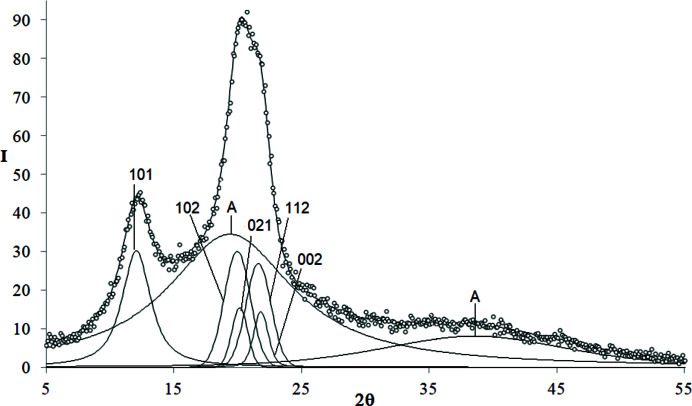
WAXD curve of cellulose II decomposed into crystalline peaks and amorphous maxima: experimental curve – points, best-fitted theoretical curve and all components – solid line.

*Cellulose II (CeII)*. The WAXD curve of cellulose II contains five crystalline peaks and two amorphous maxima (Fig. 6 ▸). The total number of unknown parameters determined with the PSO optimization procedures was 28.

**Figure 7:**
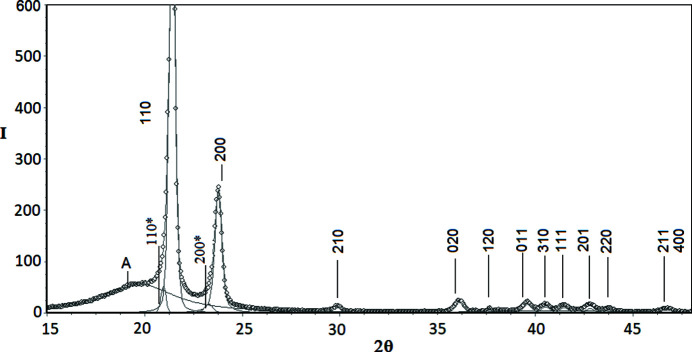
WAXD curve of polyethyl­ene (PE) sample decomposed into crystalline peaks and amorphous maxima: experimental curve – points, best-fitted theoretical curve and all components – solid line [16 crystalline peaks, two peaks, (110)* and (200)*, arising from the partly ordered phase and two amorphous halos].

*Polyethyl­ene (PE)*. In the case of the WAXD curves of PE the two strongest peaks (110) and (200) (Fig. 7 ▸) are slightly asymmetric. This means that the models in which these peaks are approximated by a linear combination of Gauss and Cauchy profiles do not give a good fit to the experimental curve. For this reason, the approach of Baker & Windle (2001 ▸) has been employed in this work.

According to these authors, the peaks (110) and (200) seem to be asymmetric because two smaller and broader peaks are localized on their left, low-angle side. Baker and Windle have proposed that these additional peaks [denoted here as (110)* and (200)*] are related to a third, partly ordered phase, the density of which is intermediate between those of crystalline and amorphous phases. Because the third phase is much less ordered than the crystalline one, it does not contribute to the remaining crystalline peaks located at higher angles (Baker & Windle, 2001 ▸). The hypothesis of Baker and Windle on the existence of the third, partly ordered phase in PE is fully consistent with the results presented by other authors (Wang *et al.*, 1991 ▸; Kitamaru *et al.*, 1996 ▸; Suzuki *et al.*, 1985 ▸; Popli *et al.*, 1984 ▸, *etc.*). Thus, the WAXD curve of PE was decomposed into 16 crystalline peaks, two peaks (110)* and (200)* arising from the partly ordered phase and two amorphous maxima. The total number of unknown parameters was 76.

The angular positions of crystalline peaks of PE determined by means of the PSO optimization procedure were identical for all five tested methods and identical to those resulting from the unit-cell parameters of PE. This is why a respective table comparing the experimental and literature data is not presented. Literature data were calculated based on the unit-cell parameters of PE given by Swan (1962 ▸).

**Figure 10:**
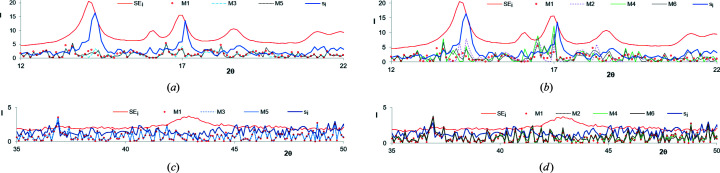
A comparison of the errors SE*_i_* and *s_i_* with the absolute values of residuals obtained in the tested methods for iPP (*a*), (*b*) in the 2θ ranges with high intensities and (*c*), (*d*) in the 2θ ranges with low intensities. The presented values were calculated using normalized WAXD curves of iPP.

*Polyethyl­ene terephthalate (PET)*. The WAXD curve of PET and best-fitted theoretical curve are shown in Fig. 10 ▸. The curve contains 12 crystalline peaks and two amorphous maxima. The total number of unknown parameters determined with the PSO optimization procedures was 64. The angular positions determined with the PSO algorithm are listed in Table 6 ▸. For the methods M1, M2 and M3 they agree well with the literature data calculated based on the unit-cell parameters of PET (Daubeny *et al.*, 1954 ▸). However, in the case of methods M4 and M6 big discrepancies between experimental and literature data are observed.

**Figure 11:**
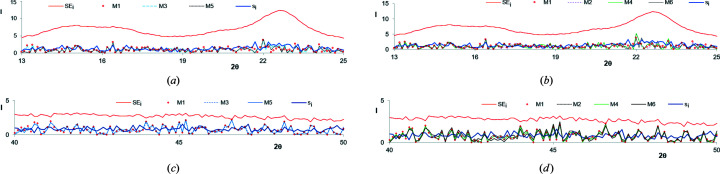
A comparison of the errors SE*_i_* and *s_i_* with the absolute values of residuals obtained in the tested methods for CeI (*a*), (*b*) in the 2θ ranges with high intensities and (*c*), (*d*) in the 2θ ranges with low intensities. The presented values were calculated using normalized WAXD curves of CeI.

*Polyamide 6 (PA6)*. The WAXD curve of PA6 and best-fitted theoretical curve are shown in Fig. 11 ▸. The curve contains five crystalline peaks and two amorphous maxima. Their angular positions determined with the PSO algorithm agree well with the literature data calculated based on the PA6 unit-cell parameters given by Holmes *et al.* (1955 ▸).

### 6.2. Analysing and testing of the residuals   

Figs. 10 ▸(*a*)–10 ▸(*d*) present a comparison of the errors SE*_i_* and *s_i_* with the absolute values of residuals obtained in the tested methods for iPP in the 2θ ranges with high intensities [(*a*) for the methods M1, M3 and M5 and (*b*) for the methods M1, M2, M4 and M6] and in the 2θ ranges with low intensities [(*c*) for the methods M1, M3 and M5 and (*d*) for the methods M1, M2, M4 and M6]. A similar comparison related to CeI is presented in Figs. 11 ▸(*a*)–11 ▸(*d*).

The figures testify that both in the 2θ ranges with high (crystalline peaks) and low values of intensity the residuals are much smaller than the standard errors SE*_i_*. No correlation, trends or interdependencies between the errors SE*_i_* and *s_i_* can be noticed.

In the case of the models obtained for the WAXD curve of iPP the residuals are also smaller than the root-mean-square errors *s_i_* calculated based on five independently recorded curves. In the case of PVDF, CeI and CeII, the residuals are very close to the respective errors *s_i_*. One can notice that the highest residuals are obtained using the methods M4 and M6. The residuals obtained in the methods M1, M3 and M5 are nearly the same – the respective plots nearly overlap each other. Similar results were also obtained for PVDF and CeII.

### 6.3. Statistical tests   

To check if the residuals obtained in a given method met the necessary conditions listed in Section 5, we performed suitable statistical tests. The hypotheses that the conditions were fulfilled were verified with the significance level of 0.05 which meant that the probability of rejection of a true hypothesis was 0.05.

**Figure 12:**
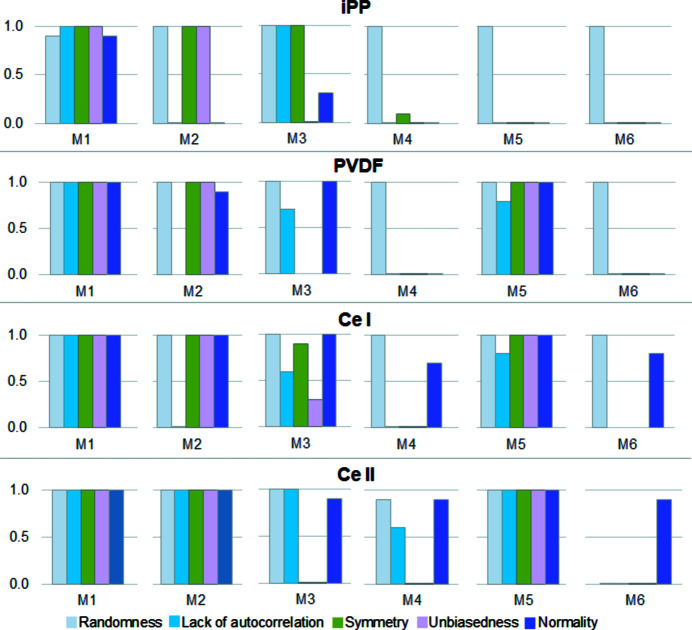
The results of performed tests for the models of the WAXD curve of iPP, PVDF, CeI, CeII.

The results of the performed tests are shown graphically in Fig. 12 ▸. As each decomposition procedure with a given method (M1–M6) was run ten times, the presented values are the fractions of positive results of a given test, *i.e.* the number of positive results of a test divided by ten. Obviously, the higher this value, the better fulfilled is the tested condition.

The plots testify that only in the case of method M1 are all conditions related to the residuals met for all polymers. The residuals in method M2 are most frequently correlated. In methods M3 and M5 they are asymmetric and biased. Moreover, the residuals in method M5 are correlated. In the case of methods M4 and M6 only the randomness of the residuals is fulfilled.

### 6.4. Testing of the homoscedasticity of residuals   

The homoscedasticity of residuals means that they are of the same order of magnitude in all subranges of the whole recording range. They cannot systematically increase or decrease with increasing diffraction angle. To verify if residuals are homoscedastic, the whole range of diffraction angles (5–60°) in which the WAXD curves were recorded was divided into four subranges. For a given polymer the subranges were chosen in such a way that one of them comprised points with the highest values of intensity (crystalline peaks) and another one included points with the lowest intensities (peripheral region). For each range the average value of residuals obtained in each method was calculated. According to the condition of homoscedasticity, the averages of residuals in all subranges should not differ significantly from each other and should be close to zero.

**Figure 13:**
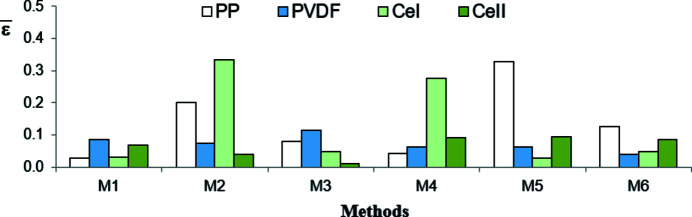
Absolute values of the averages of residuals 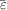 in the subranges with the highest intensity (regions with crystalline peaks).

**Figure 15:**
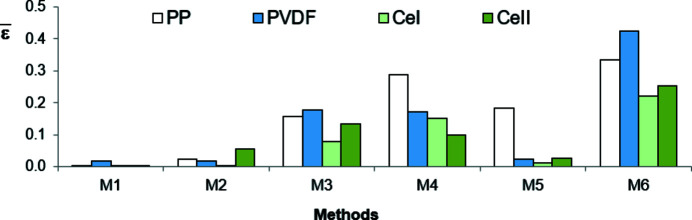
Absolute values of the averages of residuals 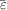 in the whole recording range.

The averages of residuals 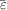 (their absolute values) in the subranges with the highest and the lowest intensities, calculated for all methods and polymers, are shown in Figs. 13 ▸ and 14 ▸, respectively. Fig. 15 ▸ shows the averages of residuals (their absolute values) in the whole recording range. As we see, only for the residuals obtained with method M1 is the condition of homoscedasticity met for all polymers. Method M2 gives small residuals in the subranges with low intensity values and big ones in the subranges of high intensity, while the inverse is true for method M3. In the case of methods M4 and M6 the residuals are big in the whole recording range and different for different polymers.

**Figure 16:**
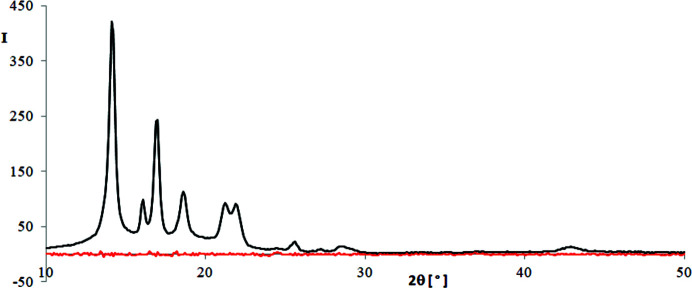
WAXD curve of iPP (black line) and residuals (red line) obtained with method M1.

Fig. 16 ▸ may serve as a good illustration of the homoscedasticity of residuals in method M1. In this figure we can compare the WAXD curve of iPP and residuals obtained with this method. The residuals are very small and nearly the same in the whole 2θ range.

### 6.5. Quality of mathematical models of WAXD curves   

**Figure 17:**
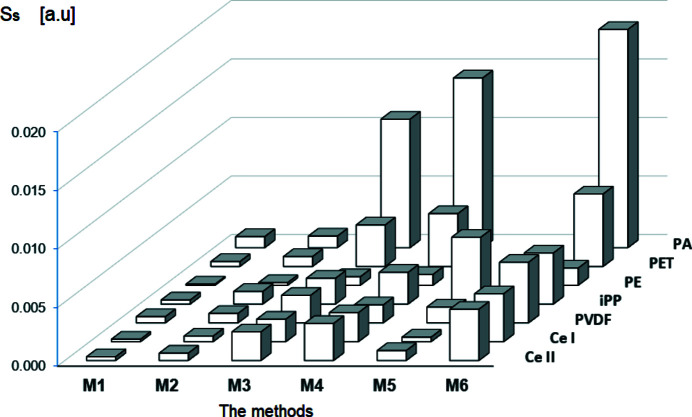
The integral similarity index *S_S_* for investigated methods.

To evaluate the quality of mathematical models of WAXD curves obtained by means of the tested methods M1–M6, the integral similarity index *S_S_* and normalized *R* index were calculated for all models and compared with each other. A graphical presentation of the obtained results is given in Figs. 17 ▸ and 18 ▸. The presented values of both indices were calculated by averaging the results obtained in ten runs of the decomposition procedure. It should be emphasized that the lower the values of *S_S_* and *R*, the better is the model, *i.e.* the better quality of fitting of the theoretical curve to the experimental one. It is clearly seen in the figures that for all polymers the lower values of analysed indices, and thereby the highest quality of fitting, is reached by method M1.

As has already been mentioned in Section 5, the quality of fitting offered by a given model can also be visually evaluated by comparing the so-called theoretical integral intensity curve 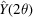 related to the model with the experimental integral intensity curve *Y*(2θ) related to the experimental WAXD curve. The integral intensities can be calculated using formulas (18), (19) and plotted in one figure. The shapes of the integral intensity curves of various polymers differ clearly from each other, being characteristic for a given polymer.

**Figure 19:**
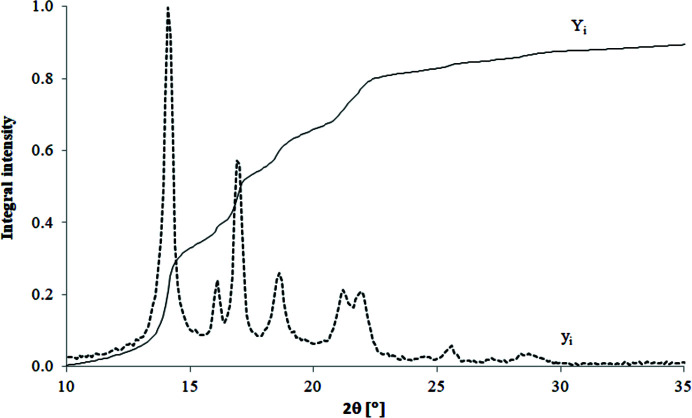
Experimental integral intensity curve calculated for iPP superimposed onto the original WAXD curve.

Fig. 19 ▸ shows the experimental integral intensity curve determined for iPP superimposed onto the original WAXD curve.

**Figure 20:**
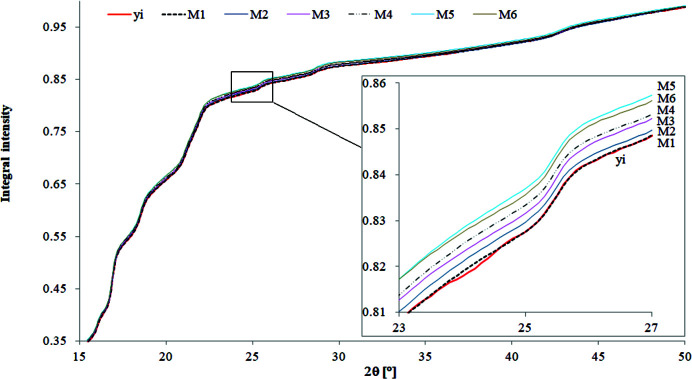
Experimental integral intensity curve for iPP (red line *y_i_*) and theoretical integral intensity curves obtained with methods M1–M6 shown in a large range of the diffraction angle 2θ: 15–50°. One can see that the experimental curve and the theoretical curve obtained with method M1 nearly overlap each other.

Fig. 20 ▸ presents the experimental integral intensity curve of iPP and theoretical integral intensity curves obtained with methods M1–M6. We see that all theoretical intensity curves are very close both to each other and to the experimental integral intensity curve. However, a magnified picture in the inset shows clear shifts between the curves. A careful inspection of such plots, performed in the whole range of the diffraction angle 2θ, showed that generally for all polymers the smallest differences between theoretical and experimental integral intensity curves are observed in the case of the models obtained with method M1.

## 7. Summary   

The analyses presented in this work clearly show that the objective function which is minimized in the fitting of a theoretical curve to the experimental one influences considerably the shape of the theoretical curve and its parameters. In comparing and evaluating the six methods of fitting of a theoretical curve to the experimental one, it was assumed that the best method should not only give the crystalline peak positions compatible with the crystalline structure of the investigated polymer but also it should be marked by the lowest values of the informational criteria *S_S_* and *R*, and the smallest differences between experimental and theoretical integral curves. Moreover, the residuals obtained with this method should fulfil the conditions described in Section 5.

The performed analysis shows that all these requirements were fulfilled in the best way by method M1 (least-squares method). Moreover, it was shown that for all tested polymers the scatter of crystallinity values obtained with this method is the smallest (Table 1 ▸). It means that in order to obtain the most credible mathematical model of an experimental curve, useful for determination of the structural parameters of the investigated polymer, we should employ this method.

The least absolute deviations method (M2) treats the tops of sharp reflections as outliers and for this reason it should not be used for polymers, the WAXD curves of which contain narrow crystalline peaks (*e.g*. iPP or PVDF). Employing this method we obtained higher values of *S_S_* and *R* and bigger differences between the integral curves. Furthermore, the residuals were not always normally distributed and uncorrelated.

Yet higher values of *S_s_* and *R* and bigger differences between the integral curves were obtained for all polymers when the weighted methods M3 (least relative squares), M4 (least absolute relative deviations), M5 (weighted least squares) and M6 (least-squared relative deviations) were used. Additionally, the residuals obtained with these methods did not meet the required conditions and usually they were biased, asymmetric and interrelated. The obtained results showed that method M6 was the worst of all compared methods.

The investigations presented in this work show that it is difficult to find rational reasons which could justify application of the objective functions based on relative errors in mathematical modelling of the WAXD curves.

## Figures and Tables

**Figure 8:**
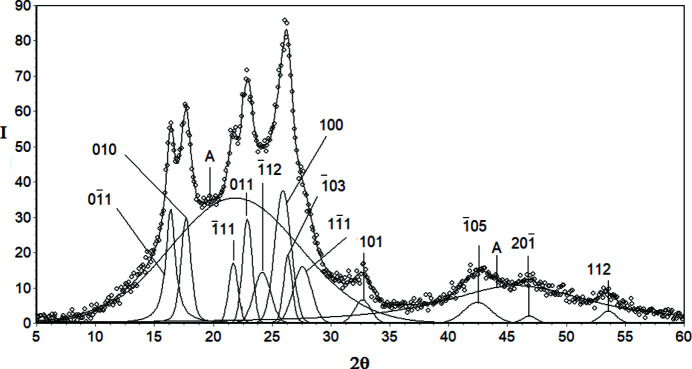
WAXD curve of PET decomposed into crystalline peaks and amorphous maxima by means of the PSO algorithm: experimental curve – points, best-fitted theoretical curve and all components – solid line.

**Figure 9:**
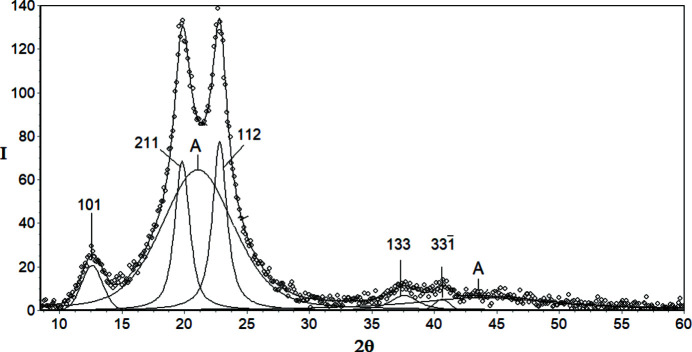
WAXD curve of PA6 decomposed into crystalline peaks and amorphous maxima by means of the PSO algorithm: experimental curve – points, best-fitted theoretical curve and all components – solid line.

**Figure 14:**
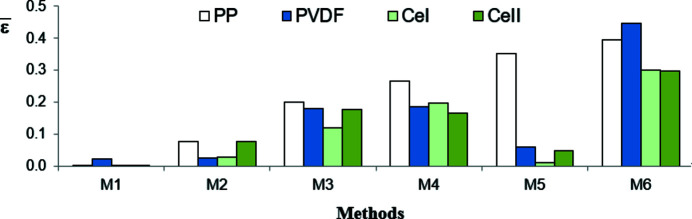
Absolute values of the averages of residuals 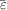 in the subranges with the lowest intensity (peripheral regions).

**Figure 18:**
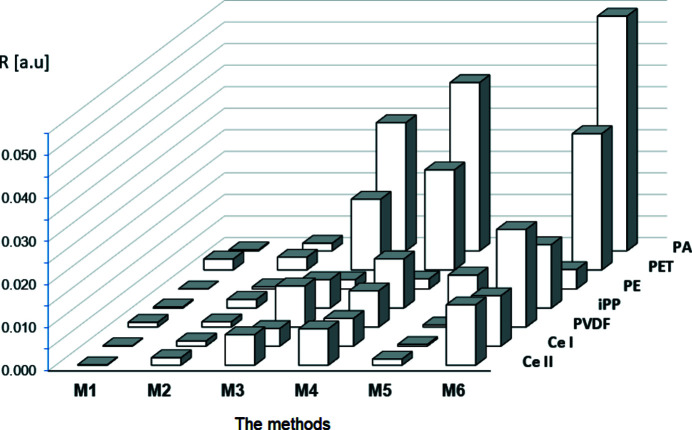
The integral similarity index *R* for investigated methods.

**Table 1:** Range of the degree of crystallinity values (%) obtained in ten runs of calculations with a given method and the biggest difference Δ*X* between the crystallinity obtained with different methods Method M5 was not employed in the case of PE, PA6 and PET.

	M1	M2	M3	M4	M5	M6	Δ*X*
PP	57–58	50–54	58–59	50–57	55–57	54–58	9
PVDF	31–32	31–34	31–33	29–33	32–33	36–40	11
CeI	46–48	48–51	46–50	45–48	47–52	47–49	7
CeII	31–33	28–30	31–34	28–31	30–31	28–29	6
PE	62–63	61–62	62	60–62	–	60–62	3
PET	30–31	33–35	34–38	32–35	–	41–51	21
PA6	35–38	25–31	29–34	23–28	–	32–33	15

**Table 2:** Angular positions of crystalline peaks of iPP determined by means of the PSO optimization procedure using methods M1–M6 The presented values were calculated as the average of the results obtained from ten runs of the decomposition procedure. In all cases, the maximum value of the standard deviation was smaller than 0.02°. Literature data were calculated based on the unit-cell parameters of iPP given by Mencik (1960 ▸).

	Miller index (*hkl*)											
	300	110	020	040	130	400	111	121	060	200	220	013
Crystalline phase	β	α	β	α	α	β	α	β	α	α	α	α
Literature data, 2θ (°)	13.9	14.1	16.1	16.9	18.5	18.6	21.3	21.7	25.5	27.0	28.4	42.5
M1, 2θ (°)	14.0	14.1	16.1	16.9	18.6	18.7	21.2	21.9	25.5	27.2	28.7	42.9
M2, 2θ (°)	14.0	14.1	16.1	16.9	18.6	18.7	21.2	21.9	25.5	27.3	28.7	42.9
M3, 2θ (°)	14.0	14.1	16.1	16.9	18.6	19.1	21.2	21.9	25.5	27.2	28.7	42.9
M4, 2θ (°)	14.0	14.1	16.1	16.9	18.6	18.6	21.2	21.9	25.5	27.3	28.7	42.9
M5, 2θ (°)	13.9	14.1	16.1	16.9	18.5	18.6	21.2	21.9	25.5	27.2	28.7	42.9
M6, 2θ (°)	14.0	14.1	16.1	16.9	18.6	18.8	21.2	21.9	25.5	27.2	28.7	42.9

**Table 3:** Angular positions of crystalline peaks of PVDF determined by means of the PSO optimization procedure using methods M1–M6 The presented values were calculated as the average of the results obtained from ten runs of the decomposition procedure. In all cases, the maximum value of the standard deviation was smaller than 0.02°. Literature data were calculated based on the unit-cell parameters of PVDF given by Lovinger (1982 ▸).

	Miller index (*hkl*)													
	010	100	020	110	200	101	021	121	130	200	210	002	230	310
Crystalline phase	α	α	α	α	β	α	α	α	α	α	α	α	α	α
Literature data 2θ (°)	9.1	17.9	18.4	20.1	20.7	26.4	26.7	32.4	33.2	36.2	37.5	39.0	46.3	56.5
M1, 2θ (°)	9.3	17.8	18.5	20.0	20.3	26.6	26.7	32.3	33.2	35.9	37.1	38.7	46.4	56.1
M2, 2θ (°)	9.3	17.8	18.5	20.0	20.1	26.6	26.7	32.6	33.2	35.9	37.1	38.7	46.5	56.1
M3, 2θ (°)	9.3	17.8	18.5	20.0	20.2	26.6	26.7	32.3	33.2	35.9	37.1	38.7	46.4	56.0
M4, 2θ (°)	9.3	17.8	18.5	20.0	20.3	26.6	26.7	32.5	33.2	35.9	37.1	38.7	46.5	56.1
M5, 2θ (°)	9.3	17.8	18.5	20.0	20.1	26.6	26.7	32.3	33.2	35.9	37.1	38.8	46.4	56.1
M6, 2θ (°)	9.3	17.8	18.5	20.0	20.2	26.6	26.7	32.3	33.2	35.9	37.1	38.8	46.3	56.0

**Table 4:** Angular positions of crystalline peaks of cellulose I determined by means of the PSO optimization procedure using methods M1–M6 The presented values were calculated as the average of the results obtained from ten runs of the decomposition procedure. In all cases, the maximum value of the standard deviation was smaller than 0.02°. Literature data were calculated based on the unit-cell parameters of cellulose I given by Ellefsen (1960 ▸).

	Miller index (*hkl*)									
	110	101	101	120	002	121	221, 130	040, 103	301	004, 104
Crystalline phase	α	α	α	α	α	α	α	α	α	α
Literature data 2θ (°)	13.9	14.7	16.6	20.4	22.7	22.7	29.2	34.9	36.3	46.3
M1, 2θ (°)	13.6	14.9	16.6	20.4	22.4	22.7	29.4	34.5	36.2	46.3
M2, 2θ (°)	13.2	14.8	16.6	20.3	22.4	22.7	29.5	34.5	36.4	46.6
M3, 2θ (°)	13.8	14.9	16.6	20.3	22.4	22.7	29.4	34.5	36.1	46.6
M4, 2θ (°)	13.6	14.9	16.6	20.3	22.4	22.7	29.4	34.4	36.1	46.7
M5, 2θ (°)	14.1	14.9	16.5	20.4	22.4	22.7	29.5	34.5	35.9	45.8
M6, 2θ (°)	13.1	14.9	16.6	20.3	22.4	22.7	29.6	34.4	36.0	47.2

**Table 5:** Angular positions of crystalline peaks of cellulose II determined by means of the PSO optimization procedure using methods M1–M6 The presented values were calculated as the average of the results obtained from ten runs of the decomposition procedure. In all cases, the maximum value of the standard deviation was smaller than 0.02°. Literature data were calculated based on the unit-cell parameters of cellulose II given by Paralikar & Batrabet (1981 ▸).

	Miller index (*hkl*)				
	101	102	021	112	002
Crystalline phase	α	α	α	α	α
Literature data 2θ (°)	12.1	19.5	20.5	21.4	22.0
M1, 2θ (°)	12.1	19.7	20.2	21.6	21.8
M2, 2θ (°)	12.1	19.7	20.2	21.5	21.8
M3, 2θ (°)	12.1	19.7	20.2	21.4	21.9
M4, 2θ (°)	12.1	19.9	20.3	21.6	22.1
M5, 2θ (°)	12.1	20.0	20.3	21.7	21.9
M6, 2θ (°)	12.1	19.9	20.1	21.6	22.0

**Table 6:** Angular positions of crystalline peaks of PET determined by means of the PSO optimization procedure using methods M1–M4 and M6 The presented values were calculated as the average of the results obtained from ten runs of the decomposition procedure. In all cases, the maximum value of the standard deviation was smaller than 0.02°. Literature data were calculated based on the unit-cell parameters of PET given by Daubeny *et al.* (1954 ▸).

	Miller index (*hkl*)											
	011	010	111	011	112	100	103	111	101	105	201	112
Literature data 2θ (°)	16.4	17.5	21.3	23.4	24.6	25.6	26.4	27.9	32.7	42.6	47.13	53.4
M1, 2θ (°)	16.4	17.7	21.7	23.0	24.3	25.9	26.3	27.5	32.7	42.5	46.8	53.6
M2, 2θ (°)	16.4	17.7	21.7	22.9	24.8	25.6	26.3	27.3	32.7	42.5	46.9	53.6
M3, 2θ (°)	16.4	17.7	21.7	22.9	24.8	25.9	26.4	27.6	32.6	42.6	46.9	53.5
M4, 2θ (°)	15.0	16.3	20.0	21.4	22.7	24.2	24.7	25.9	30.6	39.7	43.6	49.9
M6, 2θ (°)	16.0	17.3	21.4	23.0	24.2	26.0	26.3	27.7	32.7	42.6	46.9	53.7

**Table 7:** Angular positions of crystalline peaks of PA6 determined by means of the PSO optimization procedure using methods M1–M4 and M6 The presented values were calculated as the average of the results obtained from ten runs of the decomposition procedure. In all cases, the maximum value of the standard deviation was smaller than 0.02°. Literature data were calculated based on the unit-cell parameters of PA6 given by Holmes *et al.* (1955 ▸).

	Miller index (*hkl*)				
	101	211	112	133	331
Literature data 2θ (°)	12.3	19.8	22.8	37.4	40.3
M1, 2θ (°)	12.6	19.8	22.84	37.6	40.6
M2, 2θ (°)	12.6	19.8	22.86	37.6	40.6
M3, 2θ (°)	12.6	19.8	22.83	37.5	40.7
M4, 2θ (°)	12.6	19.8	22.86	37.6	40.8
M6, 2θ (°)	12.6	19.9	22.80	37.4	40.7
